# COVID-19 surveillance systems in African countries

**DOI:** 10.34172/hpp.2021.49

**Published:** 2021-12-19

**Authors:** Yusuff Adebayo Adebisi, Adrian Rabe, Don Eliseo Lucero-Prisno III

**Affiliations:** ^1^Global Health Focus Africa, Nigeria; ^2^African Young Leaders for Global Health, Abuja, Nigeria; ^3^Faculty of Pharmacy, University of Ibadan, Ibadan, Nigeria; ^4^Faculty of Medicine, School of Public Health, Imperial College London, UK; ^5^Department of Global Health and Development, London School of Hygiene and Tropical Medicine, UK

**Keywords:** COVID-19, Surveillance, Responses, Africa, Global health, Pandemic

## Abstract

**Background:** Surveillance forms the basis for response to disease outbreaks, including COVID-19. Herein, we identified the COVID-19 surveillance systems and the associated challenges in 13 African countries.

**Methods:** We conducted a comprehensive narrative review of peer-reviewed literature published between January 2020 and April 2021 in PubMed, Medline, PubMed Central, and Google Scholar using predetermined search terms. Relevant studies from the search and other data sources on COVID-19 surveillance strategies and associated challenges in 13 African countries (Mauritius, Algeria, Nigeria, Angola, Cote d’Ivoire, the Democratic Republic of the Congo, Ghana, Ethiopia, South Africa, Kenya, Zambia, Tanzania, and Uganda) were identified and reviewed.

**Results:** Our findings revealed that the selected African countries have ramped up COVID-19 surveillance ranging from immediate case notification, virological surveillance, hospital-based surveillance to mortality surveillance among others. Despite this, there exist variations in the level of implementation of the surveillance systems across countries. Integrated Disease Surveillance and Response (IDSR) strategy is also being leveraged in some African countries, but the implementation across countries remains uneven. Our study also revealed various challenges facing surveillance which included shortage of skilled human resources resulting in poor data management, weak health systems, complexities of ethical considerations, diagnostic insufficiency, the burden of co-epidemic surveillance, and geographical barriers, among others.

**Conclusion:** With the variations in the level of implementation of COVID-19 surveillance strategies seen across countries, it is pertinent to ensure proper coordination of the surveillance activities in the African countries and address all the challenges facing COVID-19 surveillance using tailored strategies.

## Introduction

The COVID-19 outbreak is a threat to public health worldwide, including African countries.^1-3^ On 30 January 2020, the outbreak was classified by the World Health Organization (WHO) as a global health emergency and it was later announced as a global pandemic on 11 March 2020.^4^ In Africa, the first COVID-19 case was confirmed in Egypt in mid-February 2020.^1^ The virus has rapidly spread making it a major health concern on the continent.^5^ As of 29 May 2021, 4 854 497 cases and 130 214 deaths have been recorded in Africa^6^ and stakeholders have continued to intensify efforts to curtail the pandemic.^4^ Due to notable weak laboratory systems in Africa, it can be surmised that there is a widespread underreporting of COVID-19 cases in many countries in Africa.^7^ Aside from the weak healthcare systems on the continent, long-standing challenges such as limited infrastructure and equipment, inadequate funding systems for health, and ineffective data transmission systems for surveillance among others, continue to impact health.^1^

Even though there has been a quick response to the outbreak on the continent well before cases were reported, the impact of the pandemic is far-reaching.^4^ In early 2020, the Africa Centres for Disease Control (CDC) and Prevention established the Africa Task Force for Novel Coronavirus.^8^ The task force has been working with the WHO African Region (AFRO) on risk communication and community engagement, surveillance, infection control and prevention in health care facilities, clinical management of COVID-19 patients, and laboratory diagnosis.^8^

According to the WHO, COVID-19 surveillance involves monitoring and reducing the transmission and spread of the disease through a systematic collection, analysis, and interpretation of data as well as timely dissemination of analyzed data in order to understand the patterns and size of the pandemic.^9^ The WHO also recommends active surveillance, with a focus on early case finding, aggressive testing, and contact tracing in all possible transmission cases.^9^ COVID-19 surveillance aims to reduce the spread of disease and provide the opportunity for national health authorities to curtail the COVID-19 pandemic to ensure early resumption of social and economic activities across countries.^10^ Effective surveillance is also recommended to track COVID-19 transmission trends and the virus dynamics as the pandemic evolves.^10^

African countries need to strengthen COVID-19 surveillance capacities to quickly identify, and care for COVID-19 cases as well as to trace and quarantine their contacts and monitor the trend of the disease over time.^4^ Robust national surveillance will require the adaptation and strengthening of pre-existing national systems, where needed, and the revitalization and implementation of additional surveillance capacities, as appropriate.^10^ At the beginning of the pandemic, based on the huge number of travels and direct links to China, the WHO identified 13 priority African countries for COVID-19 - Mauritius, Algeria, Nigeria, Angola, Cote d’Ivoire, the Democratic Republic of the Congo, Ghana, Ethiopia, South Africa, Kenya, Zambia, Tanzania, and Uganda.^11^ Currently, no review article comprehensively analyzed the COVID-19 surveillance and the challenges facing it. Hence, this study aimed to:

1. Identify and catalogue the COVID-19 surveillance systems in the 13 WHO-identified COVID-19 priority countries in Africa.
2. Describe the challenges facing COVID-19 surveillance in the 13 WHO-identified COVID-19 priority countries in Africa.

## Materials and Methods

We conducted a narrative review of data sources to identify the COVID-19 surveillance and associated challenges in 13 African countries i.e., Mauritius, Algeria, Nigeria, Angola, Cote d’Ivoire, the Democratic Republic of the Congo, Ghana, Ethiopia, South Africa, Kenya, Zambia, Tanzania, and Uganda. The narrative review was conducted to summarize COVID-19 surveillance strategies in 13 African countries as it was surmised that few studies answer our research question based on a preliminary search of studies. We also believe that this independent review of surveillance systems for COVID-19 in African countries will provide insights on the topic.

A comprehensive search in Medline, PubMed, PubMed Central, and Google Scholar using search terms including “Surveillance”, “case finding”, “contact tracing” “surveillance response system” “case reporting” “early detection” “monitoring”, “tracking”, “early warning”, “COVID-19” 2019-nCoV, “SARS-CoV-2” “{+Each African Countries - Mauritius, Algeria, Nigeria, Angola, Cote d’Ivoire, the Democratic Republic of the Congo, Ghana, Ethiopia, South Africa, Kenya, Zambia, Tanzania, and Uganda}.

The inclusion criteria include data sources that provide information regarding COVID-19 surveillance systems in the predetermined African countries and were published in English between January 2020 and April 2021 while the exclusion criterion was any other data sources that do not provide information regarding COVID-19 surveillance systems in the 13 African countries. Two members of the research team (YAA and AR) who are skilled health systems researchers were involved in the literature review to gather data for this study independently. The collected articles were managed using Endnote Reference Manager Software version X8 with a prior review of the title and abstract. In case of any duplication of references or disagreement, a consensus was reached through discussion. Additionally, to ensure the quality of data and correctness of facts (check and balance), we also informally reached out to stakeholders in the African countries for secondary verifications. The authors of some selected papers were also reached out to.

The researchers also snowball further data sources i.e., relevant articles were gotten from reference lists of the articles identified through our systematic search. Supplementary data were also gathered from country reports, newsletters, commentaries, policy briefs and other reports as well as direct google search as it was inferred that some of the relevant sources are not published in peer-reviewed academic journals because they are not empirical studies but policy papers. The extracted data were discussed narratively to explore the aim of the study.

## Results and Discussion

### Category of COVID-19 surveillance systems

To effectively curb the pandemic, WHO released a guidance document in December 2020 which epitomizes public health surveillance of COVID-19.^9^ This document supersedes and combines the two previously released documents, which are Global Surveillance Guidance for COVID-19 Caused by Human Infection with COVID-19 Virus: Interim Guidance, and Surveillance Strategies for COVID-19 Human Infection: Interim Guidance. This combined report showed that surveillance systems can be melded across different sites (See Table 1). The surveillance systems are contact tracing, immediate case notification, virologic surveillance, mortality surveillance, cluster investigations, and event-based surveillance, as well as participatory surveillance, serologic surveillance and surveillance using telephone hotlines. The report also identified various sites for surveillance which include community, primary care sites, hospitals and sentinel sites, enclosed settings (e.g., prisons, dormitories, and long-term living facilities), health care centers associated COVID-19 infection, and travelers at point-of-entry. In this study, we used the framework from the interim guidance on the COVID-19 surveillance systems by WHO to describe the strategies and approaches used in the 13 African countries.

**Table 1:** WHO category for surveillance across various site and context

**Site/context**	**System**					
	**Immediate case notification**	**Contact tracing**	**Virologic surveillance**	**Cluster investigations**	**Mortality surveillance**	**Serologic surveillance**
Community	×	×		×	×	×
Primary Care Sites (non-sentinel ILI/ARI)	×		×	×		
Hospitals (non-sentinel ILI/SARI)	×		×	×	×	×
Sentinel ILI/ARI/ SARI sites	×		×			
Closed settings (prisons, dormitories, and long-term living facilities)	×	×		×	×	×
Health care-associated SARS-CoV-2 infection	×	×		×	×	×
Travelers at Points of Entry	×	×		×		

We summarized the COVID-19 surveillance approaches and mechanisms in the 13 African countries in Table 2. We also described different surveillance systems below.

**Table 2:** Summaries of COVID-19 surveillance systems in the 13 African countries

**Country**	**System**								
	**Immediate Case Notification**	**Contact Tracing**	**Virological Surveillance**	**Mortality Surveillance**	**Serologic Surveillance**	**Event-Based Surveillance**	**Genomic Surveillance**	**Environmental Surveillance**	**Participatory Surveillance**
Algeria	×	×	×	×			×		×
Angola	×	×	×	×	×		×	×	×
Nigeria	×	×	×	×	×		×	×	×
South Africa	×	×	×	×	×	×	×	×	×
The Democratic Republic of Congo	×	×	×	×	×		×		×
Zambia	×	×	×	×	×		×		×
Kenya	×	×	×	×	×		×	×	×
Uganda	×	×	×	×		×	×		×
Tanzania					×			×	
Ethiopia	×	×	×	×	×	×	×		×
Mauritius	×	×	×	×			×		×
Ghana	×	×	×	×	×	×	×		×
Cote d’Ivoire	×	×	×	×	×		×		×

*Immediate case notification:*involves an early reporting of cases to national health authorities, usually within 24 to 48 hours to enhance response activities and reduce the spread of the virus in all sites and contexts.^9,12^ Immediate case notification is a critical approach to ensure adequate and early response activities are formulated to address the pandemic.^10^ WHO also recommends routine surveillance for case notification which include: comprehensive (testing all suspected cases), case-based (reporting probable and confirmed cases within 48 hours of identification and outcome reporting within 30 days of the first report), or aggregate (weekly reporting) routine surveillance.^9^

Case notifications can involve getting data from both active and passive surveillance.^10^ Some of the sources of data for COVID-19 case notification and ascertainment include laboratory reporting, clinician reporting, direct reporting by a patient to health authorities, and reporting by other entities (e.g., schools, hospitals, laboratories, pharmacies, veterinarians, airports, etc.) among others.^12^ Most of the 13 African countries including Nigeria,^13^ Zambia,^14^ and South Africa^15^ had strategies to ensure immediate case notification of COVID-19 suspect, probable and, confirmed cases (as well as syndromic early-warning surveillance, using instantaneous, often a wide-ranging symptom or initial diagnosis information collected during the usual healthcare provision) to national health authorities. However, data sharing regarding COVID-19 cases in Tanzania revealed that case notification is not a priority as the country battle chronic COVID-19 denialism during the era of late President John Magufuli.^1^ Partner organizations e.g., Centers for Diseases Control Tanzania continues to support surveillance in the country.^16^ On 6 April 2021, the new President Samia Suluhu Hassan pushed for a change in approach from her predecessor, proposing an evaluation of the country’s response to COVID-19 in order to ensure an evidence-based approach, and a return to regular publishing of data.^17^

*Contact tracing:*has been a pillar of infectious disease control in public health for many years. It involves spotting on persons who may have come in contact with an infected person and successive collection of further information about these contacts.^10^ According to WHO, in the context of COVID-19, contact is referred to as a person that has direct or person-to-person contact with a confirmed or probable case, usually within one meter for a minimum of fifteen minutes with/without the use of personal protective equipment.^18^

Contact tracing is an important approach for disrupting chains of transmission of coronavirus and curtailing COVID-19-associated deaths.^18^ Many African countries (e.g. Democratic Republic of Congo) with their experiences in handling previous outbreaks, such as the Ebola virus, have developed best practices for contact tracing and this has been rebranded for the pandemic response.^19^ Some countries e.g. Nigeria have also integrated contact tracing with well-structured systems and networks for infectious diseases case finding.^20^ African countries are also investing in capacity building of healthcare workers, students, and volunteers in order to ensure effective contact tracing.^21^ A review study has also shown that there are about 186 contact tracers per 100 000 population in Uganda, 3 contact tracers per 100 000 population in South Africa, and an average of 111 contact tracers per 36 states in Nigeria.^21^ Most countries in Africa continue to strengthen the contact tracing systems in order to ensure effective COVID-19 response.^21^

*Laboratory-based surveillance:* includes both virologic surveillance and serologic surveillance.^10^ Virologic surveillance is an important part of the COVID-19 surveillance system as it allows the national health authorities to understand the coronavirus in order to curb the pandemic.^9^ At the beginning of the pandemic, the nucleic acid amplification test (e.g., *reverse transcription polymerase chain reaction*) was the only WHO-recommended (reference standard) method for confirmation of a case or clusters that must be reported with 24 hours.^9^

WHO also recommends that using the clinical specimens that are obtained through national sentinel surveillance of ILI (*influenza*-like illness) or ARI(acute respiratory infection) or SARI (severe acute respiratory infection) using RT-PCR.^9^ Later into the pandemic, WHO introduces antigen-detecting rapid diagnostic tests as a confirmational method which is simpler, less expensive, less sensitive, and faster to perform than the reference standard (*reverse transcription polymerase chain reaction*).^9^ Virological surveillance is also important in identifying clusters of cases (2 or more COVID-19 *cases* associated with the same group, location, or event around the same time) in order to formulate an immediate response. Most African countries continue to strengthen their testing capacity in order to ensure effective virologic surveillance.^4^ By the end of 2020, all African countries can test for COVID-19 using RT-PCR with the support of WHO, national health authorities, and other stakeholders.

Serosurveillance is also critical in supporting the investigation of the COVID-19 outbreak and understanding the size of the outbreak.^22,23^ It involves detecting antibodies produced by the human body in response to COVID-19.^23^ Serosurveillance studies and efforts have also been reported in some African countries e.g Nigeria,^24,25^ Côte d’Ivoire,^23^ South Africa,^26,27^ Kenya,^28,29^ Ethopia,^30^ and other sub-Saharan African countries.^31,32^

*Mortality surveillance:*involves reporting and monitoring the number of COVID-19 deaths in the community, hospitals including long-term facilities, and other sites.^33^ In the context of COVID-19, the number of deaths must be recorded daily or within a week.^10^ WHO defined a COVID-19 death for surveillance purposes as a death resulting from a clinically compatible illness in a probable or confirmed COVID-19 case, unless there is a clear alternative cause of death that cannot be related to COVID-19 (e.g., trauma cases).^9^ Efforts to conduct COVID-19 mortality surveillance have been seen in African countries and this is evident in the daily/weekly reporting of COVID-19 mortality in the region.^34-36^

*Hospital-based surveillance:*is a form of facility-based surveillance that takes place within the hospital and daily zero reporting of cases is pertinent to ascertain the effectiveness of the surveillance systems.^10^ It also involves monitoring suspected and confirmed COVID-19 cases among healthcare workers in the hospital.^9^ Patients with probable or confirmed COVID-19 admitted to hospitals should be notified to health authorities within 1 day of identification and the outcome should not be delayed in terms of reporting once it is available.^9^ Most African countries continued to strengthen COVID-19 surveillance in hospitals.^37-41^

Others forms of surveillance include:

*Event-based surveillance:* involves monitoring, assessing, organized collection, and interpretation of mainly unstructured information usually from formal, and informal channels such as radio and TV broadcasts, community online content, and print media regarding health events or risks.^42^ The implementation of event-based surveillance will make for a strengthened capacity to rapidly detect any changes in the overall COVID-19 situation.^43^ Africa CDC has been aggregating event-based surveillance data reported by the African Union Member States since January 2020, well before the first cases on the continent were reported.^44^ WHO has also reported that event-based surveillance can be useful in humanitarian settings and other resource-limited settings.^9^ Efforts to strengthen event-based surveillance have been ongoing in some African countries and further intensified with the emergence of the COVID-19 pandemic e.g. Surveillance Outbreak Response Management and Analysis System (SORMAS) in Nigeria and community event-based surveillance in Tanzania.^16,45-47^

*Genomic surveillance:* involves a systematic, active, and regular collection of genetic sequence information from a pathogen population to develop an effective response.^48^ In the COVID-19 context, it involves identifying mutations that could affect how the coronavirus functions or spreads.^48^ Detecting variants of pathogens and setting up a public health response to them requires a robust genomic surveillance system.^49^ Genome sequencing of the coronavirus is important for tracing the sources and perhaps for drawing lessons to prevent future outbreaks.^49^

**Figure 1:**
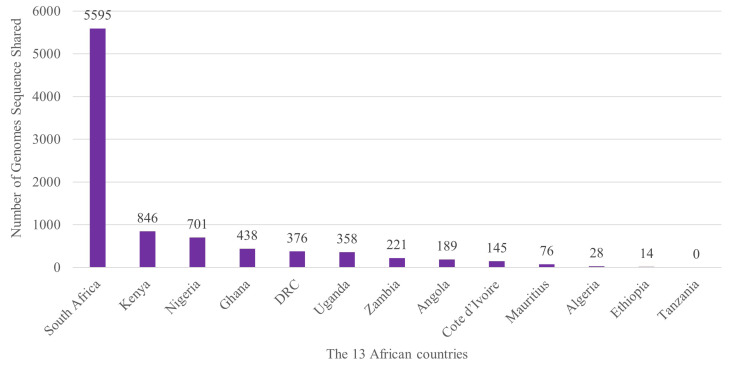
The number of genomes sequence shared from 10 January 2020 to 12 May 2021 in the 13 African countries.

Since the emergence of the COVID-19 pandemic, many African countries have published their country sequences.^50^ For instance, three days after the confirmation of the index COVID-19 case in Nigeria, the genome sequencing results of the SARS-CoV-2 specimen were published on 1 March 2020.^51^ Making Nigeria the first country in Africa to publish the genomic sequence due to the collaboration between Nigeria Center for Diseases Control, the Nigeria Institute for Medical Research and the Africa Centre for Excellence in Genomics (ACEGID).^51^ ACEGID has the most developed capacity and is also a reference laboratory for the joint WHO and Africa CDC COVID-19 Genome Sequencing Laboratory Network.^51^ In Figure 1, we revealed the number of genomes sequence shared from 10 January 2020 to 12 May 2021 in the 13 African countries, according to data from GISAID - Global Initiative on Sharing Avian Influenza Data.^52^

*Environmental surveillance:* has a long-standing use in public health and it involves testing of wastewater (sewage) for the presence of diseases causing organisms e.g., wild poliovirus, and more latterly in antimicrobial resistance surveillance.^53^ In the context of the present COVID-19 outbreak, it is being utilized for the detection of the presence of the coronavirus shed into wastewater.^54^ According to WHO, infectious coronavirus has not been detected in wastewater, which implies transmission via sewage contamination is unlikely.^55^ However, WHO recommends wastewater surveillance has an optional and complementary approach to COVID-19 surveillance in addition to other indicator-based surveillance.^55^ Several studies have recommended wastewater-based surveillance for Africa and other low-income nations because of the indiscriminate widespread open defecation near water sources and the presence of untreated sewage close to homes.^56-59^ Community monitoring of coronavirus in wastewater can therefore prove useful as a surveillance tool.^55,58-60^ Despite the benefits of wastewater-based surveillance for COVID-19, it requires skilled laboratory staff and costly equipment to manage sophisticated sample processing and handling.^61^ This presents the reason most African countries have not implemented wastewater-based surveillance for COVID-19.^62,63^

*Participatory surveillance:* is a form of surveillance in public health that involves active engagement and empowerment of communities and individuals by leveraging digital platforms, telephones, mobile applications, and other media to self-report symptoms and other important information regarding a health threat.^64^ Participatory surveillance is also a means to modernize the process of monitoring and tracking outbreaks and to ensure early implementation of measures to effectively contain them.^64^ For instance, SORMAS, a digital early warning and disease management system that was developed during the 2014-2015 Ebola outbreak by public health experts in Germany and Nigeria, is increasingly adapted for use in integrating various diseases surveillance in a user-friendly manner.

In the context of COVID-19, it has been shown that the use of participatory surveillance to supplement conventional health surveillance systems provides a quick way for the data collection on symptomatic individuals in communities.^65^ There is a growing interest in Africa to implement participatory surveillance systems in response to COVID-19. For instance, digital platforms and mobile apps have been developed in Nigeria,^66^ Ethiopia,^67^and Kenya^68^ to facilitate case reporting, symptoms reporting, and risk communication.Data gotten through participatory surveillance can also clarify changes in health-seeking behaviour while interpreting facility-based surveillance data.^9^

As revealed in Table 2, there exist variations in the level of implementation of the COVID-19 surveillance across the 13 African countries. Most countries continue to engage in participatory surveillance, virological surveillance, mortality surveillance, early case notification, genomic surveillance and, contact tracing for COVID-19 while event-based surveillance and environmental surveillance are least implemented. Our findings also revealed that South Africa’s surveillance strategy is widespread, and this is probably responsible for the fact that the country has the highest number of COVID-19 cases in Africa. Tanzania’s responses to COVID-19 have been worrisome and this is clear from how poorly the country has developed COVID-19 surveillance strategies.

### Integrated disease surveillance and response for COVID-19 in Africa

In order to address the issue of weak diseases surveillance on the African continent, the WHO AFRO published the maiden technical guideline for Integrated Disease Surveillance and Response (IDSR) strategy and began to implement it in 1998.^69^ The strategy provides a framework for reaching the 2005 International Health Regulations core capacity requirements to strengthen disease surveillance system at the national level.^69^ The second edition of the IDSR framework strategy was released in 2010 and the third edition was published in 2019.^70^ The overall aim of the strategy was to enhanced surveillance and response capacities at each level of the national health systems in order to facilitate early responses to public health emergencies.^70^ The strategy also makes it possible to integrate a number of surveillance systems, and link laboratory and surveillance data to guide response activities.^71^ During the first year of the emergence of COVID-19, the IDSR strategy assisted in investigating, identifying, and responding to outbreaks of circulating vaccine-derived type 2 poliovirus, measles, yellow fever, hepatitis virus, and COVID-19 in many locations.^71^

Many African countries continue to struggle to implement IDSR.^70^ Recently, the WHO AFRO released a report to encourage national health authorities to strengthen IDSR for COVID-19.^72^ Prior to the pandemic, the implementation of IDSR across African countries is uneven – while some countries IDSR is performing well, it remains poorly implemented in others.^73^ With the variation in the level of implementation, the IDSR system has been leveraged for COVID-19 response in many African countries including the Democratic Republic of Congo and Nigeria among others.^74^ It has allowed for intensive surveillance(case-based and syndromic) as well as entry point for characterizing, identifying, and responding to person-to-person transmission of COVID-19, where it is fully implemented.^74^ Overall, surveillance needs to be strengthened, based on IDSR guidelines, with enhanced index of suspicion for cases.

Similar to longstanding challenges facing IDSR implementation in Africa, the overall COVID-19 surveillance systems are not without challenges on the continent ranging from poor data management, limited resources, and funding to shortage of skilled staff among others.^70^ The challenges facing COVID-19 surveillance were discussed extensively in the next section of the manuscript.

### Challenges facing COVID-19 surveillance in Africa

We identified challenges facing COVID-19 surveillance in the selected African countries which are also applicable to other African countries.

*Shortage of human resources*

This is a long-standing challenge facing effective surveillance in Africa.^70,73,75-77^ There are various tasks in diseases surveillance that requires expertise in other to ensure effective response e.g., data analysis, early case reporting, and understanding surveillance data use in response measures.^70,77^ This fundamental challenge also continues to undermine COVID-19 surveillance e.g., genomic surveillance and environmental surveillance in some African countries.^62,63,77^ The challenge of limited skilled staff is also doubly compounded by the incessant brain-drain surge in Africa.

The shortage of skilled staff has also contributed to poor surveillance data management and substandard transmission of data for effective surveillance.^70,77^ Even though African government and other partners have been making efforts to organize training to address the issue of lack of skilled human resources, more still needs to be done in order to strengthen COVID-19 surveillance expert pool.^70,72^

*Stigma and misinformation*

In the COVID-19 context, there are a growing number of evidence of social stigmatization against healthcare workers, people that are previously affected by COVID-19, and areas with a high incidence of the disease.^78,79^ The stigmatized people/areas are often being stereotyped, separated, labelled, and/or experience loss of status and discrimination because of the untoward affiliation with the disease, which is further fueled by misinformation (infodemic).^80^ Some of the hoaxes in some African countries include “The COVID reports are fabricated”, “Young people are immune to the pandemic”, “Communities without soap say maybe they can use very hot water for washing hands”, “People say masks are not meant for them but for a particular class of people and race”, “It’s a strategy to get rid of Africans”, “COVID-19 is cured by drinking ginger tea”, “It is a disease of the politicians”, “The people involved are just doing it for money’s sake”, “COVID is for the rich”, and “God is annoyed with humans because they have abandoned Him by stopping to gather for worship” among others.^1^ All these can contribute to a lack of cooperation by the populace for proper surveillance.

The misinformation is propagated by word of mouth but also accelerated by social media, which has a much wider reach, and more rapid dissemination functions.^1^ The social stigma and misinformation associated with the pandemic continue to make case finding, disease tracking, and contact tracing difficult and this is doubly compounded by social media. For instance, in some African countries e.g., Nigeria,^81^ South Africa,^21^ and Uganda,^21^ the fear due to misinformation and stigma attached to COVID-19 has limited the number of people who show up for testing or report symptoms and also make contact tracing challenging. Inertia and resistance to COVID-19 response measures are also contributing to ineffective surveillance on the continent.^1^ Africa needs to continue to strengthen risk communication and community engagement as well as social media surveillance to address the social stigma and infodemic.^1^

*Diagnostic insufficiency*

COVID-19 testing capacity has increased from two national laboratories in February 2020 to about one thousand laboratories in early 2021 across the African region and availableness of rapid antigen diagnostic tests.^72^ Despite this, increasing testing to the required level of 10 per 10 000 population remains a challenge across the region.^72^ Many African countries are experiencing community (person-to-person) transmission, yet 31 out of the 46 countries reported fewer than 10 tests per 10 000 people per week in March 2021.^82^ This implies that the number of cases reported in the region may not truly reflect the COVID-19 situation on the continent. This is more concerning because it is important to count all COVID-19 cases including probable cases.^9^

In addition to this, the delay in confirmation due to limited quantities of COVID-19 testing kits also contributes to weak surveillance on the continent.^72^ Experts have reported that the low number of cases in Africa is due to poor surveillance as a result of low testing rates.^7^ Some of the challenges facing COVID-19 testing in Africa include insufficient human resources, limited laboratory resources and infrastructure, and logistics constraints.^7,83^ Laboratory surveillance remains an essential strategy to understand the size of the pandemic on the continent. It is important to strengthen the testing capacity (including non-laboratory-confirmed clinical cases) in Africa in order to ensure early case reporting for necessary response activities.

*Burden of co-epidemic surveillance*

The need to respond to existing infectious and non-infectious diseases contribute to inadequate COVID-19 surveillance in Africa.^4^ For instance, Nigeria has to ensure Lassa fever surveillance,^84^ the Democratic Republic of Congo needs to monitor Ebola outbreak,^85^ and Ethiopia has to contain the cholera outbreak,^86^ amid COVID-19 pandemic. Africa is facing a high burden of many diseases and it is important to formulate measures to respond to them without disrupting COVID-19 surveillance. This further reiterates the need for African countries to implement an effective IDSR for disease surveillance on the continent.^72^

*Complexities of ethical consideration*

Some surveillance strategies, especially those that involved the use of digital technologies, are faced with complexities of ethical considerations.^87,88^ Many African countries are already implementing participatory digital apps for COVID-19 contact tracing, reporting of symptoms, and other surveillance parameters.^66-68^ The meddlesome nature of this approach is concerning, especially for public health and legal ethics.^88^ For instance, in Nigeria^89^ and South Africa^90^ concerns have been raised regarding the use of digital tools for surveillance. It is therefore important that African countries implement effective legislation that will promote digital rights, privacy, and data protection to ensure accountability and transparency of the surveillance data. It is also important for countries to abide by WHO guidelines for ethical consideration in using digital technologies for surveillance.^91^

*Geographical barriers*

Uneven access to healthcare services in Africa due to geographical barriers may also pose barriers to COVID-19 surveillance in Africa.^1,5^ The World Bank reported that about six out of every ten individuals in Sub-Saharan Africa are an inhabitant of rural areas.^92^ COVID-19 response activities are limited in rural Africa due to lack of resources and poor road networks among others.^5^ This challenge facing COVID-19 response on the continent does not spare surveillance.^93^ The use of contact tracing apps and other digital surveillance tools are nearly impossible in rural communities where access to smartphones and electricity is a challenge.^5^ Even with the use of human contact tracers, it is challenging to reach some areas due to poor road networks and other geographical barriers such as a large bodies of water, and mountains, which obstruct ease of access for case finding in remote places.^94^ Overcrowding in resource-poor settings with deficient access to sanitation serves significant challenges for contact tracing, case isolation, and finding, which are important measures for reducing transmission.^93^ Additionally, sample collection and transportation may also be challenging in far-to-reach/hard-to-reach areas of Africa.^94^ This also serves as a challenge to surveillance in humanitarian settings. It is therefore important that countries developed tailor-made strategies e.g., the use of mobile laboratories, to ensure surveillance in remote places are not affected.

*Weak healthcare systems*

The need torevitalize and strengthen diseases surveillance in Africa is long overdue.^2,21,95^ However, due to a lack of political will to improve the healthcare system, resources to response to public health emergencies are often limited.^95^ Weak COVID-19 surveillance is linked to a long-standing lack of investment in to the healthcare system.^96^ This is evident in how many African government expenditures on health always falls short of the Abuja Declaration, where at least 15% of the total budget should be allocated to health.^95^ For instance, many African countries rely on donors for test kits and other laboratory resources,^97^ which is not sustainable for effective laboratory surveillance in the long run. Some African countries do not have the capacity to conduct genomic and environmental surveillance due to a lack of resources.^63,98^ Low health workforce and limited health facilities are also contributing indirectly to weak COVID-19 surveillance in Africa.^98-100^ It is therefore important that the African government should continue to combat this defiance in order to improve the overall healthcare systems on the continent.

Our study is not without its limitations. First, our review does not include some databases such as Web of Science and Scopus. However, the databases used in the manuscript provided critical insights on the topic and will add value to strengthen COVID-19 surveillance and surveillance to other public health emergencies in Africa. Second, there is a paucity of extensive data regarding the strategies used for COVID-19 surveillance in Africa. Hence, the need to extend our search to the use of grey literature such as government reports, websites, and newsletters among others. Third, only papers published in English are included. Even though most of the COVID-19 papers are published in English, there is a possibility that we may have excluded some studies in local languages. Fourth, the unsystematic search method used in the study may lead to the subjective selection of articles and consequently add bias to the overall interpretation of findings. Authors considered this limitation in their search strategy, but with limited published evidence in the literature, they prioritized the need for an overview of this selected topic.

## Conclusion and recommendations

Our findings revealed variations in the level of implementation of COVID-19 surveillance strategies in the 13 African countries because of many reasons and determinants. It is important to ensure proper coordination of the surveillance activities in the African countries. National health authorities should reflect and use the emergence of COVID-19 to rethink the need to strengthen diseases surveillance, not just for now but for future public health emergencies. Surveillance remains a key pillar for effective response to diseases epidemics. We recommend that countries should continue to implement IDSR and address all the challenges facing COVID-19 surveillance using tailor-made strategies in order to ensure effective COVID-19 response. Investment in quality data management systems and capacity building of operational team on data analysis and interpretation of surveillance data is pertinent.

## Acknowledgements

We thank Global Health Focus for the supervision, expert advice, contact with countries and other supports.

## Funding

YAA is a recipient of 2020 Royal Society of Tropical Medicine and Hygiene and National Institute of Health Research UK Small Grant Award. The authors undertake this work with the funding support from Royal Society of Tropical Medicine and Hygiene and National Institute of Health Research UK Small Grant Award.

## Competing interests

The authors declare no conflict of interest.

## Ethical approval

As a narrative review, additional approval from ethical committee was not applicable.

## Authors’ contributions

YAA led, conceptualized, and wrote the paper. YAA performed the literature review with the support from AR. DELP and AR supervised and critically reviewed the manuscript. All authors have read and agreed to the final version of the paper.

## Disclaimer

The authors claim that no part of this paper is copied from other sources.
